# *In vivo*, noncontact, real-time, PV[O]H imaging of the immediate local physiological response to spinal cord injury in a rat model

**DOI:** 10.1117/1.JBO.25.3.032007

**Published:** 2019-10-25

**Authors:** Seth Fillioe, Kyle K. Bishop, Alexander V. S. Jannini, John J. I. Kim, Ricky McDonough, Steve Ortiz, Jerry Goodisman, Julie Hasenwinkel, Charles M. Peterson, Joseph Chaiken

**Affiliations:** a Syracuse University, Department of Chemistry, Syracuse, New York, United States; b Syracuse University, Syracuse Biomaterials Institute, Department of Chemical and Biomedical Engineering, Syracuse, New York, United States

**Keywords:** spinal cord injury, PV[O]H, turbidity, hematocrit, *in vivo*, imaging

## Abstract

We report a small exploratory study of a methodology for real-time imaging of chemical and physical changes in spinal cords in the immediate aftermath of a localized contusive injury. One hundred separate experiments involving scanning NIR images, one-dimensional, two-dimensional (2-D), and point measurements, obtained *in vivo*, within a 3×7  mm field, on spinal cords surgically exposed between T9 and T10 revealed differences between injured and healthy cords. The collected raw data, i.e., elastic and inelastic emission from the laser probed tissues, combined via the PV[O]H algorithm, allow construction of five images over the first 5 h post injury. Within the larger study, a total of 13 rats were studied using 2-D images, i.e., injured and control. A single 830-nm laser (100-μm diameter round spot) was spatially line-scanned across the cord to reveal photobleaching effects and surface profiles possibly locating a near surface longitudinal artery/vein. In separate experiments, the laser was scanned in two dimensions across the exposed cord surface relative to the injury in a specific pattern to avoid uneven photobleaching of the imaged tissue. The 2-D scanning produced elastic and inelastic emission that allowed construction of PV[O]H images that had good fidelity with the visually observed surfaces and separate line scans and suggested differences between the volume fractions of fluid and turbidity of injured and healthy cord tissue.

## 1 Introduction

On every level, the costs of spinal cord injury (SCI) are staggering.^1^ We devote tens of billions of dollars each year in addressing the societal costs and that is small compared to human suffering on the individual level. We can presently do little to affect the eventual outcome for the injured person in terms of functionality and subsequent quality of life. In this paper, we very briefly present a new algorithm, i.e., the PV[O]H algorithm, our procedures for collecting *in vivo* data from rats, and the results of an exploratory study to introduce PV[O]H as an imaging modality for rat spinal cord. We performed line scans to explore this possibility and observe the effect of the probing light on the tissue. Finally, we will present spinal cord images constructed from two-dimensional (2-D) scanning of an 830-nm laser across the region of interest. At each position in the scan, the PV[O]H algorithm is applied to remitted collected light, calculating (1) the apparent volume fraction of the probed tissue filled by fluid and/or fluorescent materials and (2) the apparent turbidity of that fluid.

The depth of the probed volume is roughly 300  μm when the tissue is fingertip skin with a 3% to 5% volume fraction of blood perfusion and the blood has a hematocrit (Hct) of roughly 15. The depth is greater than that for spinal cord probing with 830-nm light. Raman spectra show that we sample at least some space containing cerebrospinal fluid (CSF) but we cannot be sure how deeply we penetrate the actual neural tissues, i.e., cord below the arachnoid space. Because of elastic scattering, light propagation is less when red blood cells (RBCs) and protein are present in the fluid. We suggest the tissue depth sampled is greater for spinal cord probing than for perfused fingertip skin because (1) healthy CSF has nearly no protein in sharp contrast to plasma and (2) the perfused neural tissue, i.e., gray matter, is anatomically deeper than the less perfused white matter.

Although only a small study, we are encouraged that successive images of the same region can be obtained with our current methodology and that improvements are certainly possible. Whether or not the cord is injured, such images may vary systematically immediately after an injury. This study suggests that PV[O]H may be a valuable new imaging modality for diagnosing and treating SCI. In Sec. 1, we first present relevant issues within the specific context of SCI and then we introduce the current technology for SCI imaging and the goals we would like to address with PV[O]H imaging that are not met by other modalities.

### 1.1 Spinal Cord Injury

Although there are different kinds of spinal cord injuries, in a generic sense, all spinal cord injuries begin with an unintentional physical event that disrupts cell membranes and tissue structures and causes materials that are foreign in uninjured tissue to contact and mix with healthy tissue and fluids.^2^ Immediate cell death of neurons, glial cells, and endothelial cells occurs due to the mechanical trauma locally, defining the site of injury. This research investigates the chemical and physical state of injured spinal cord beginning within the first half hour of injury and extending to the subsequent 5 h. During this primary or immediate phase of SCI, a cascade of chemical and biological processes within the region of the injury is initiated and, regardless of the injury, the situation increases in complexity.

The secondary phase involves movement/migration of materials/cells in and out of the injured region and can last for hours to days. Note that the spatial distribution of the materials/cells during this phase may reflect the spatial distribution of the initial unintentional physical event and change over time. Observing the chemistry of injured cord tissue at the very beginning of the cascade might provide the best opportunity to contrast injured tissue with healthy tissue because healthy CSF is relatively low in protein relative to plasma. Protein produces significant background elastic and inelastically scattered light that may obscure scattering from SCI associated materials/cells. Thus, one main motivation for this research was to determine what spectroscopic information may be accessible during the immediate or primary phase. Any chance of success diagnosing and possibly treating SCI without physical contact depends on our capacity to detect and characterize chemical processes in turbid and delicate materials.

In addition to blood flow, which also distributes materials to/from the injured region from/to other parts of the body, our choice of timescale is comparable to that of the passive transport of molecules in the fluid media, i.e., CSF that fill the interstitial spaces within a healthy spinal cord. We hope that observing and possibly enumerating and identifying primary processes might suggest tactics and strategies to arrest a sequence that if left unattended will eventually form a glial scar. Alleviating scarring in turn could permit attempts at rehabilitating an injured spinal cord and promote healthier outcomes. Being able to predict that a glial scar will or will not form given the condition of a contused cord would itself be very useful in helping to decide, as soon as possible after SCI, whether desperate measures can or should be taken that entail no greater risk to the patient than doing nothing.

### 1.2 Imaging and SCI

If the initial effects of the injury reduce blood flow into and out of the injured region, the resulting hypoxia will cause additional damage to the affected tissues.^3^ PV[O]H^4^ is simultaneously sensitive to (1) the presence/absence of blood and (2) the oxygenation state of the hemoglobin and so would seem to be advantageous. Other techniques will almost certainly be applicable to addressing related issues such as blood flow, e.g., Doppler-based optical techniques or optical coherence techniques.^5^ Flow-based measurements have been made in other parts of the body, e.g., femoral artery in the context of SCI but not in the cord itself. We address some of the practicalities of trying to apply optical techniques directly to the cord in the immediate aftermath of injury. Note that the possible utility of low level laser therapy (LLLT), also known as photobiomodulation (PBM) at the earliest times might also be coupled with and benefit from this exploratory study.^6^

Previously,^2^ we described the chemistry and the subsequent chemical and physical events of SCI by studying *ex vivo* spinal cords in a rat model over a 4-day, 2-week, and 8-week post injury timescale. Near-infrared (NIR) probing revealed enhanced fluorescence that was associated with the injury. Thus, we might hypothesize that *in vivo* excitation of fluorescence during laser scanning might reveal the progression of the injury because PV[O]H has a well-defined response when fluorescence increases and decreases.

We suspect that diagnosis and treatment of SCI will probably involve many existing tools and techniques.^7^ Magnetic resonance imaging, computerized tomography, and x-rays are noncontact imaging modalities that give a three-dimensional, relatively high resolution view of the injured tissue without the need to surgically expose the cord, but none of these provide chemical information. Very high-resolution ultrasound (VHRUS) also produces clear images *in vivo* that are particularly rich in information regarding blood, but the cord must be exposed, in physical contact with a transducer, and physically/spatially stabilized in order to avoid motion defects. As for any technique *in vivo,* we have the inevitable blood flow and respiration-induced movement, and while it still provides valuable information concerning structural tissue damage and perfusion, or lack of perfusion, VHRUS does not contain chemical information. Even more invasive techniques in use today for research^8^ that may someday form the basis for an approach for treatment might benefit from real-time measures of physiological chemical signaling, i.e., real-time fluorescence changes. A major difficulty in attempting to obtain chemical information spectroscopically *in vivo* stems from the turbidity of even healthy spinal cord tissue.

In attempting to perform noninvasive *in vivo* spectroscopic probing for blood and tissues analysis^9^ in skin, we needed to deal with the turbidity of biological materials in general. To this end, we developed “PV[O]H” to quantify intravascular blood volume and composition changes. To obtain such information, one needs to deal simultaneously with both the spectroscopy and the propagation of light in the system, i.e., the turbidity. To date, we have validated PV[O]H in human and rat model studies involving skin, bacterial cultures in various media, and in unambiguous inanimate model systems.^10^^–^^13^ The meaning/nature of the quantities calculated using PV[O]H depends critically on context. When applied to skin that has a capillary vascularization, PV[O]H calculates changes in the hematocrit (Hct) and total vascular volume of the capillary network.

It is essential to emphasize that in the context of imaging spinal cords with PV[O]H we are not suggesting that we are measuring blood when we calculate “apparent Hct.” We shall label the spinal cord images produced using values calculated by PV[O]H with the word “turbidity”, which is understood to be caused by the movement/migration of materials/cells in and out of the CSF in/near the injured region. The cells can be, e.g., T-cells or other lymphocytes and the materials can be, e.g., proteins such as immunoglobulins and other large molecules that scatter light elastically appreciably or perhaps fluorescence. Other types of molecules/materials, i.e., cellular debris, would have the same effect so more information may be required to identify these cells/materials. To this end, we note that NIR probing can be done simultaneously to implement PV[O]H while obtaining Raman spectra.

The algorithm itself is very simple and rests on assumptions that are met by the problem at hand, i.e., probing spinal cords *in vivo*. But the interpretation of the results of applying this algorithm is not as if it were being applied to capillary blood in skin. PV[O]H is extremely sensitive to (1) changes in elastic scattering and/or (2) changes in fluorescence/Raman yield for any reason in any tissues. Chemical information can often be inferred from fluorescence measurements utilizing endogenous or exogenous fluorophores.^14^^,^^15^ PV[O]H can be used to produce images based on perfusion, Hct, fluorescence, or Raman spectra thereby potentially providing a new noncontact imaging modality not currently met by any other modality.

### 1.3 PV[O]H Algorithm

**Fig. 1:**
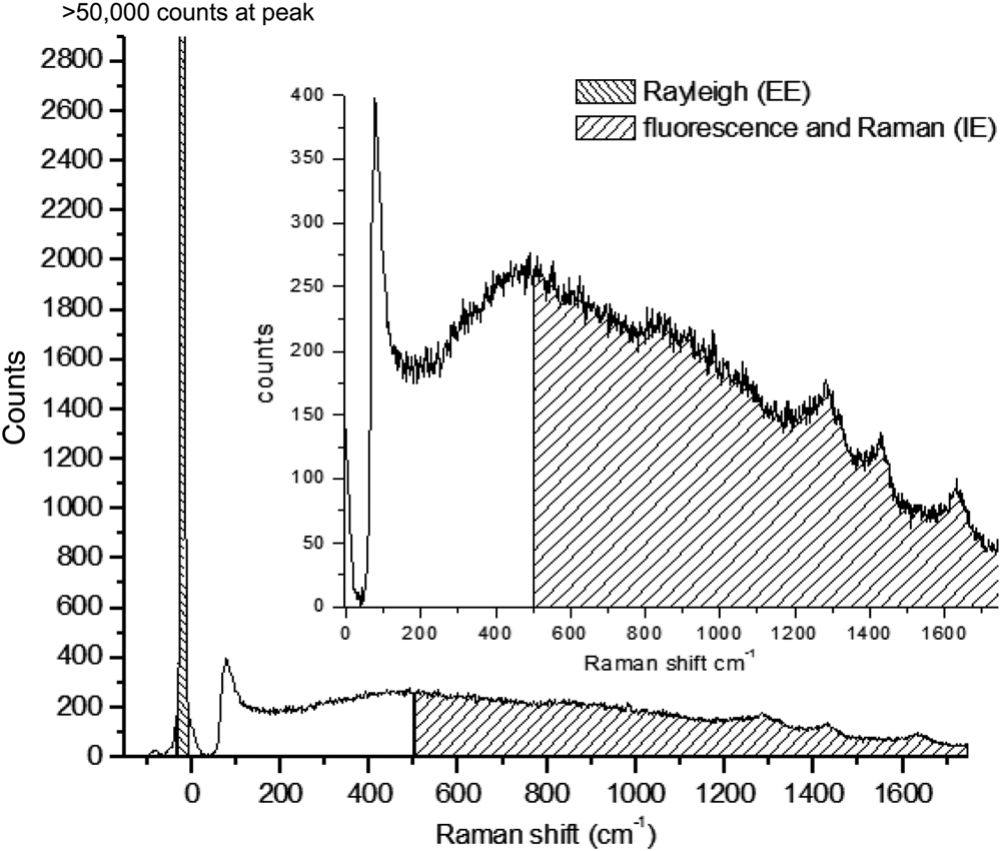
Inset: raw typical single 20 ms frame of CCD output using 200 mW of 830 nm excitation on a human fingertip. Bottom: same frame showing sections of emission integrated to estimate inelastic emission (IE, ≈500 to $1750\;{\mathrm{cm}}^{-1}$) and elastic emission (EE, −30 to $+10\;{\mathrm{cm}}^{-1}$).

Probing nearly any biological material with NIR laser light produces a remitted spectrum roughly as shown in Fig. 1. All the light that is collected caused by probing with incident NIR light has been either elastically scattered (EE) or is remitted with a wavelength shift, and so we call it inelastically scattered (IE). IE contains both Raman scattered light, phosphorescence, and fluorescence, and we use both together as shown in Fig. 1. EE and IE are produced by two fundamentally different processes, and so equations that describe their observation are independent.

The volume fractions for RBCs, plasma, and “static tissue,” which we take for everything else in the cord, sum to unity implying that there are no voids. This is summarized in Eqs. (1) and (2) using ϕ for each of the volume fractions, i.e., RBCs, plasma, and static tissue in the probed volume:

$$1=\phi_{r}+\phi_{p}+\phi_{s},$$ (1)

$$0=d\phi_{r}+d\phi_{p}+d\phi_{s}.$$ (2)

We used the radiation transfer equation (RTE)^7^ to propagate the incident light from air into a three phase, three layer medium in the single scattering limit and with the phases, i.e., the RBCs, plasma, and static tissue distributed homogeneously in the probed volume. We implicitly assume that RBCs and plasma inside the cord volume have the same optical properties as those freely circulating elsewhere. Since in skin we were able to obtain agreement with experiment by summing contributions to IE and EE linearly, and the scattering coefficients for skin are within a factor of 2 for those of cord tissue, and still at least one order of magnitude less than that for RBCs, we expect that we can write two independent equations:

$$\mathrm{EE}=\vartheta_{1}+\vartheta_{2}\phi_{p}+\vartheta_{3}\phi_{r},$$ (3)

$$\mathrm{IE}=\vartheta_{4}+\vartheta_{5}\phi_{p}+\vartheta_{6}\phi_{r}.$$ (4)

From the definition of hematocrit (Hct), we have:

$$\mathrm{Hct}=\phi_{r}/(\phi_{r}+\phi_{p}).$$ (5)

The six parameters in Eqs. (3) and (4) can be determined using the RTE and published scattering and absorption coefficients. Presently, our interest is better served by noting that since we have two linearly independent equations linking two measured quantities, EE and IE, to the two volume fractions, $\phi_{r}$ and $\phi_{p}$, for each simultaneous measurement of EE and IE, it is possible to invert Eqs. (3) and (4) to obtain

$$\varphi_{r}=a+b(\frac{\mathrm{EE}}{{\mathrm{EE}}_{0}})+c(\frac{\mathrm{IE}}{{\mathrm{IE}}_{0}}),$$ (6)

$$\varphi_{p}=d+e(\frac{\mathrm{EE}}{{\mathrm{EE}}_{0}})+f(\frac{\mathrm{IE}}{{\mathrm{IE}}_{0}}).$$ (7)

There are six parameters (a,b,c,d,e, and f) for which we must obtain numerical values. We can do this using constraints based on empirical data or assumptions. ${\mathrm{EE}}_{0}$ and ${\mathrm{IE}}_{0}$ are values obtained at the start of probing and all subsequent variation of EE and IE are with respect to these values. Thus, the apparent values of “$\phi_{r}$” and “$\phi_{p}$” so obtained are with respect to some arbitrary choice of starting values. In the present case, the device was calibrated in such a manner that the starting reference point is Hct=28.65 and all figures and graphs presented herein will reflect that choice. Since we are interested in changes in the cords over time, the choice of starting point in this methodology exploratory study is arbitrary at this point.

The experimental apparatus we used for this work was previously calibrated empirically by monitoring capillary blood in human fingertip skin *in vivo* using dialysis-induced blood composition changes independently monitored by an FDA- approved gold standard device called the CritLine. The CritLine analyzes blood inside the dialysis machine to obtain a value for Hct and thereby $\phi_{r}$ and $\phi_{p}$.

## 2 Experimental

The Institutional Animal Care and Use Committee (IACUC) of Syracuse University approved our protocol in compliance with National Institute of Health (NIH) guidelines. We performed all surgical procedures in a sterilized surgical suite located in the Laboratory Animal Research (LAR) facility at Syracuse University. We purchased 13 female Sprague Dawley rats, at weight range 250 to 330 g, from Charles River Laboratories and housed in LAR at least 2 weeks prior to surgery to acclimate to their environment. Unless specified, we obtained all materials from Thermo Fisher.

We anesthetized animals using a procedure standardized in LAR for rat surgery with approval by a certified veterinarian. After placing the animal into a sealed chamber, 5% isoflurane (Shopmetvet, Mettawa, Illinois) is allowed to flow for 2 min until animal responses are minimal. After initial anesthetization, the animal is removed from the chamber and a nosecone is used to continually flow 2% or 2.5% isoflurane throughout the procedure depending on the weight of the animal. The surgical area is shaved and sterilized using alcohol- and betadine-soaked pads. A hot water therapy pump (Braintree Scientific, Braintree, Massachusetts) was used to regulate the temperature of the animal throughout the procedure and was placed at 37°C. The device was turned on prior to anesthetization to allow adequate time to reach temperature. We sterilized all surgical tools using a microglass bead tabletop autoclave for 2 min per manufacturer instructions to ensure sterility before, during, and for cleaning after surgery.

Locations of T8, T9, T10, and T11 are verified through touch along the animal spine as both T8 and T11 spinal processes are more pronounced than surrounding vertebrata. A final check ensures deep anesthetization by tail pinch and blink reflex tests as detailed in IACUC requirements with additional tests performed to ensure continued anesthesia throughout the procedure. An ∼1-in. long incision using a #10 scalpel blade (Fine Science Tools, Foster City, California) is made along the spinal column from T8 to T11. Using surgical scissors (Fine Science Tools, Foster City, California), a hole is cut into the fat layer beneath the skin and blunt dissection is used to separate the muscle and fat layers; the fat layer is then cut and moved away from the surgical area. In parallel, incisions are made through each of the three muscle layers on either side of the spine. Once more, the position of the spinal processes is used to verify the location of T9 and T10 by moving the back of the scalpel along the vertebral column; incisions are made perpendicular to the spine between T8 and T9, T9 and T10, and finally T10 and T11.

At this point, muscle is removed in a piecemeal method using both surgical shears and scalpel to expose bone. The dorsal layer of bone for T9 and T10 is removed to expose the spinal cord to a full length of at least 1 cm using Friedman–Pearson Rongeurs. (Fine Science Tools, Foster City, California). The area was cleaned using a saline spray and sterile cotton balls prior to scanning. After completion of the experiment, all animals were humanely euthanized under anesthesia through an overdose of 0.5-ml pentobarbital (Sigma-Aldrich) by intraperitoneal injection. Verification of euthanasia was determined by cessation of respiratory functions and a blue tint to the skin. Subjects were then stored in a 20°C biohazard storage chest until offsite removal.

Directly after surgery, excess fluids in the region adjacent to the exposed spinal cord were absorbed/drawn into sterile tissue paper. We observed very light redness on the spinal cord that we attributed to irritation caused during removal of dorsal section of the vertebral column. We noted that over the succeeding 10 to 20 min, the redness faded in both controls and injury samples after initial Raman scans. During surgery, we observed no significant changes in spinal cord morphology due to Raman laser exposure or exposure to environmental conditions. A small number of small body spasms occurred, resembling simple autonomic reactions due to the anesthesia that we considered inconsequential. Fluid buildup was sometimes apparent in both control and injury samples possibly from an unknown source, although at least some was pooled saline used to hydrate the tissue that we were unable to drain away. Direct tests showed that the fluid, i.e., saline did not affect the Raman signals or show any signs of detrimental effects on the surrounding tissues.

The injury model for a rat and all techniques for creating a contusion injury were developed by Rutgers University’s W.M. Keck Center for Collaborative Neuroscience division. The contusion injury for all injured animals was produced using the Multicenter Animal Spinal Cord Injury Study (MASCIS) Impactor model III using the standard 3-mm size impaction tip at 12.5 mm above the spinal cord. Previous work with the impactor has demonstrated that the drop distance used for this study is capable of replicating a moderate injury that was believed to be sufficient for modeling a contusion injury for scanning purposes.^2^

We employed a modified commercial Raman spectrometer (Lambda Solutions, Waltham, Massachusetts) to perform all spectroscopic measurements. The optics and filtering were standard for Lambda Solutions probes with the addition of an additional Raman notch filter (Semrock, Rochester, New York) placed between the collimating lens and the grating to allow adjustment of the EE and IE for optimum dynamic range in the PV[O]H calculation. Unlike our previous published *in vivo* experiments on rats,^11^ which utilized paws, these experiments employed a standard Raman normal incidence probe having a focal length of 1 cm and an effective NA of ≈0.13 and the smallest spot size was ≈100  μm. The entire surgical field and in particular the point where light contacts the tissue was kept moist in order to prevent burning during extended exposures. The surface was contacted directly with 80 mW of CW light at 830 nm and the raw CCD data (Critical Link, LLC, Syracuse, New York) comprised 20 ms frames sampled at 50 Hz. A black tarp placed over the animal, stage, and impactor ensured as little exterior light as possible reached the Raman detector.

**Fig. 2:**
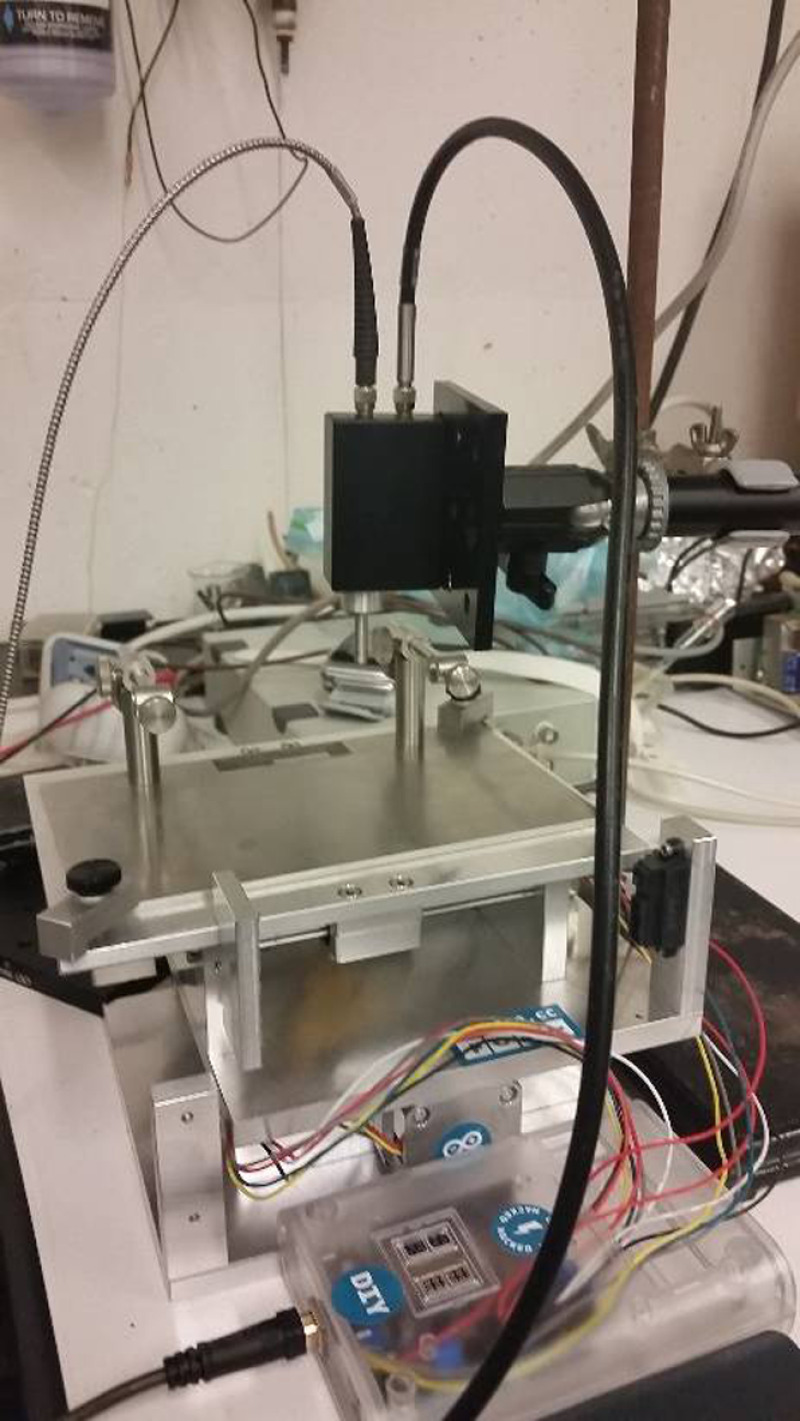
Diagram showing the X–Y stage with Raman laser above the animal clamps. The black box represents the Raman laser housing, with the silver cable supplying the laser and the black cable connecting to the detector.

In order to scan across the spinal cord of the animal, a programmable X–Y stage was developed in the Hasenwinkel lab shown in Fig. 2. An Arduino microcontroller, using custom-built MATLAB script, was capable of controlling two separate stepper motors independently with steps as small as 10  μm. An additional immobile arm held the Raman laser probe stationery while allowing the animal to be moved beneath the laser probe. Two vertebral clamps attached to the top of the stage minimized animal movement during scanning. Both the stage and the MASCIS impactor were on an immobile cart with black drapes placed on struts to prevent external light from contaminating the data.

To verify weight-drop location on the cord, the top of the stage was modified to include a rail that extended out underneath the MASCIS impactor. The laser probe was placed to minimize the diameter of the laser spot on the cord surface by visual inspection at the beginning of the experiment, before the animal is moved along the rails to underneath the weight drop. Three positions were chosen at this time along the spinal cord with reference to where the impact of the weight would occur. The first position on the cord, denoted as position A, was located toward the rostral end of the animal relative to position B, which was located at the position where injury would be induced, i.e., on the center of the weight drop. Position C was located toward the caudal end of the animal relative to position B, the position of injury. In addition, both positions A and C were chosen 1-mm off center of the spinal cord to avoid potential overlap with underlying bone while also maintaining a roughly 1 mm distance away from the predetermined position B. The animal was returned along the rail to the stage and secured once the positions were determined. We verified the laser spot size and location by observation before beginning each scanning process, i.e., when placed at position A. Since the spot size is a function of the distance between cord surface and the laser probe aperture, this check insured that the animal height did not change through the experiment.

We performed >95 separate experiments with many of the early experiments intended to explore the phenomenology and technique of probing spinal cord tissue with laser light so that practical protocols could be developed for producing scanned images. In addition to heating and possible burning when dehydrated, all biological materials remit autofluorescence that bleaches, i.e., decreases over time with continuous probing.^11^^,^^12^ In order to produce meaningful images, it is required that all tissues be probed with the same net fluence so that they are all equally bleached. Based on these observations, we designed protocols to reveal and ultimately minimize these effects on the images, both 2-D and line scans.

**Fig. 3:**
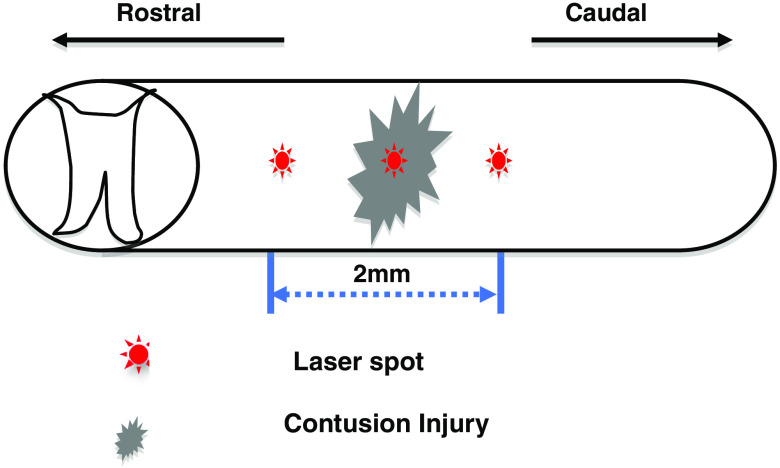
Schematic diagram depicting positioning of reference locations used to compare injured cord to healthy cord.

To compare injured tissue to healthy tissue using line scanning, one must maintain equal scanning times for each of the three positions along the spinal cord shown in Fig. 3. Also, to avoid potential burning from prolonged exposure, a set maximum scan time of <25  min was determined for one complete linear scan and 37 min for a 2-D scan. Within this total exposure timeframe, we initially placed the laser spot on position A and collected EE and IE for 5 min. The stage would then move continuously for five additional minutes while we collect EE and IE along a straight line between positions A and B. Upon reaching position B, the stage would stop while we collected EE and IE for 5 min. Afterward, scanning followed a straight-line path from position B to C. At position C, 5 min of stationery data collection ended the line scan. Thus, data at A, B, and C are the average of 15,000 CCD frames while 150 frames are averaged for each point in between corresponding to 3 s per point. This scanning process from A to B to C required 25 min to complete with the laser on continuously during the protocol. Afterward, we blocked the laser to avoid extra bleaching while the stage returned to the initial position. We monitored the animal to ensure adequate hydration of the injury site as respiration, and anesthesia were maintained throughout the procedure. This procedure was completed a total of 10 times for all control animals and for two of the injury animals with one injury animal having to be removed early due to a lack of oxygen remaining in the supply for the anesthesia device.

**Fig. 4:**
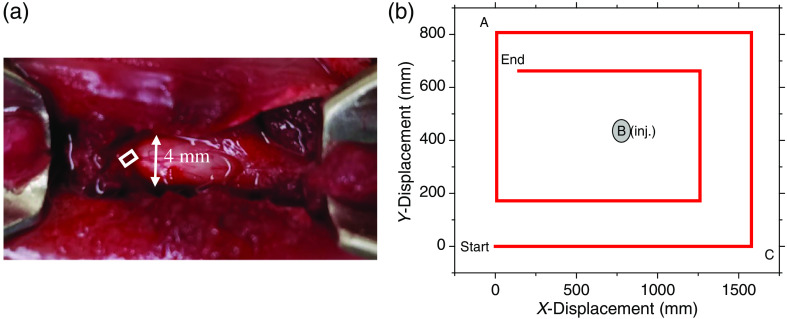
(a) Surgical field showing location of laser 2-D scanning, i.e., white box. (b) Mapping of Raman probe scanning pattern to collect data that produced the 2-D PV[O]H images of the spinal cord. Reference positions A, B, and C defined in Fig. 3 with respect to line scanning are shown in the white box and the photograph.

The 2-D scanning required special care to avoid fluorescence bleaching induced by the probing laser from defining the nature of the image. Once a scan was started, the animal was translated continuously as EE and IE were collected so the laser time at one location was minimized. Each data point, i.e., pixel was the average of 1800 20 ms CCD frames corresponding to a spatial resolution of ≈250  μm. Thus, we produced images based on the Hct values calculated at 64 points centered on different x and y locations and a scan required 37 min. The scanning pattern ensured a small overlap of adjacent pixels in both directions. Because of the fluorescence bleaching, to obtain these points, we designed the scanning pattern shown in Fig. 4(b) that did not expose any one location on the cord surface to more than one probing per image. Images can be based on the EE, IE, plasma volume, RBC, or Hct and since the scanning can be repeated, time lapsed monitoring is possible. Using the same scanning pattern for successive images results in each of the 64 positions used to produce an image having endured the same total laser fluence. When based on the EE alone, the images are essentially optical profilometry but when combined with the IE via PV[O]H, we have images based on (1) the spectroscopic properties of the materials within a depth of 300 to 500  μm of the cord surface and (2) the topography of the surface being scanned.

**Fig. 5:**
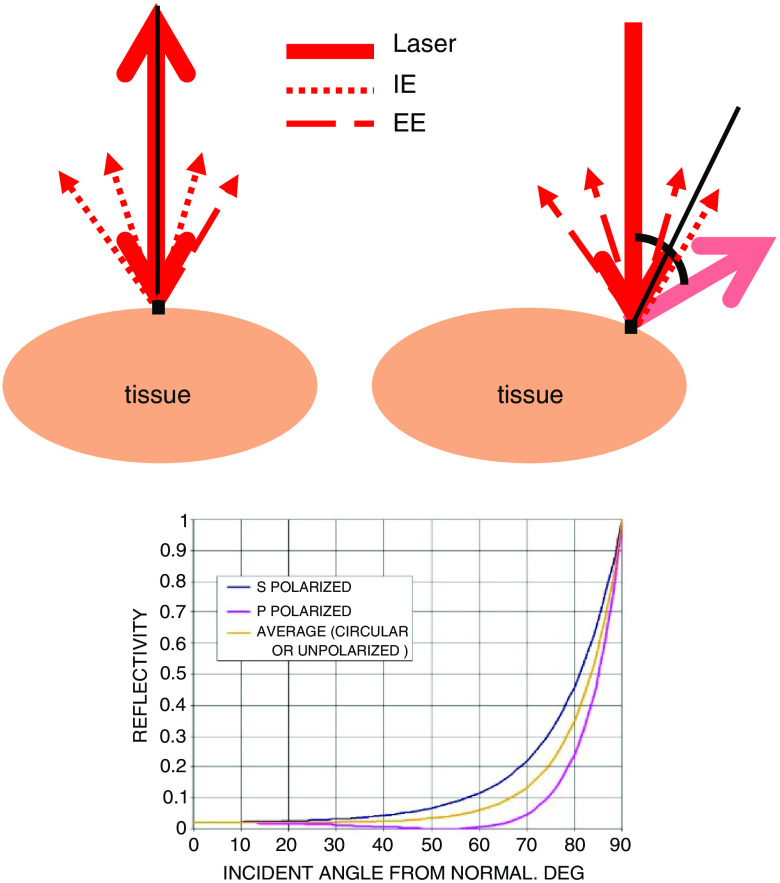
(a) Schematic (transverse view) diagram of spinal cord and probing geometry showing variation of angle of incidence with surface topography. (b) Reflectance of smooth water at 20°C (refractive index 1.333). Credit: public domain.^16^ Reflected light contributes to EE collected and light that transmits through the spinal cord dura produces both EE and IE as indicated. The mutual variation of the EE and IE between spatial locations leads to PV[O]H contrast in the image relating to surface topography.

The origin of topographical contrast is the interplay of reflection and transmission at the scanned surface. Whether line scanned or two-dimensionally scanned, PV[O]H images have fidelity with the physical appearance of the cord surface for the following reasons. When the probing light is initially brought into contact with a cord surface, a direction is defined as normal to the surface at that location. Then, when the animal is moved under the probing light to probe, the surface at different positions, e.g., as in Fig. 4, the angle of incidence may change if the surface topography changes as depicted in Fig. 5.

As can be seen in the standard reflectance curve for light traversing a water air interface, also in Fig. 5, the amount of light traversing the surface, i.e., not being reflected, varies with angle of incidence. To generate IE, the light must traverse the surface and when it does, IE is produced/detected along with EE and the apparent Hct calculated by PV[O]H manifests that transmission. This leads to contrast that reveals the surface topography. We note that the 2-D images all show a gradation of apparent Hct variation at the edges where visual inspection shows that all cord surfaces have a greater curvature near the edges.

## 3 Results

### 3.1 Line Scans

**Fig. 6:**
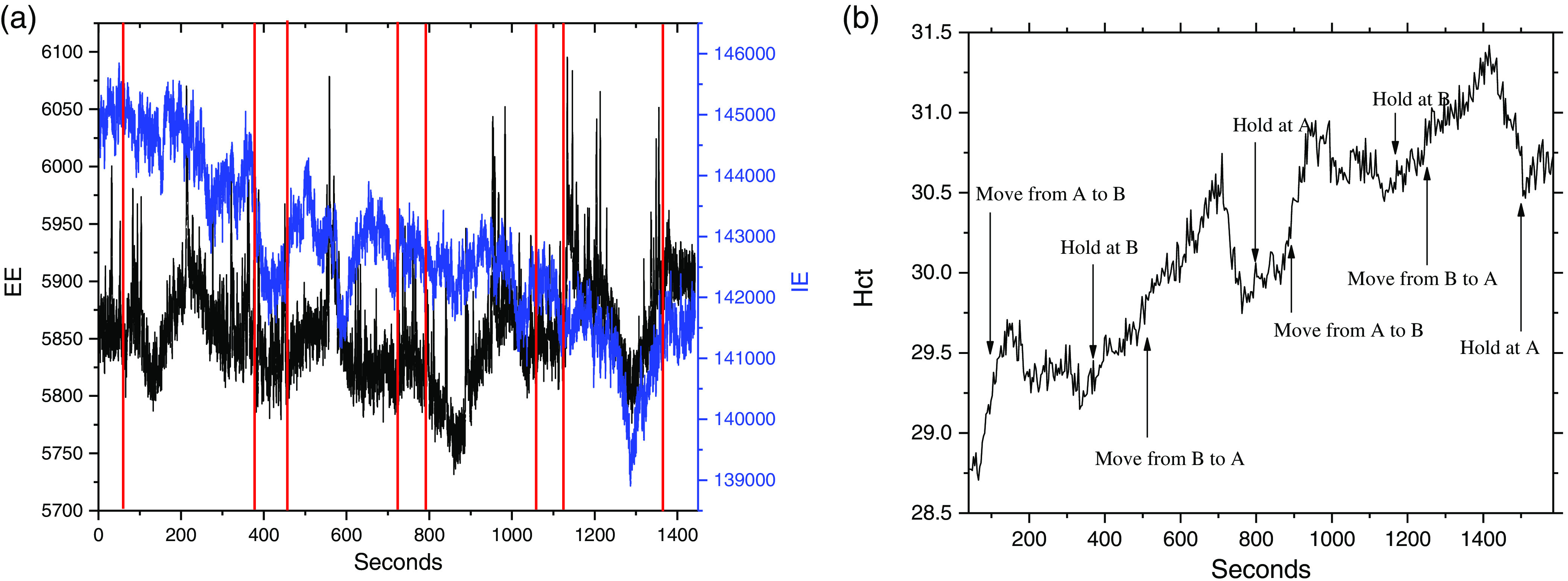
(a) The EE and IE obtained while scanning a cord for the purpose of showing how this imaging works. This scan did not follow the protocol described in Sec. 2. In this case, the scan started at A with the rat motionless for 80 s, then scanned to B in 200 s, stayed at B for 80 s, scanned back to A in 200 s, stayed at A for 80 s, rescanned from A to B in 200 s, stayed at B for 80 s, rescanned back to A in 200 s, and collected last 80 s at A. The laser probing was at the location indicated in (b): (1) at beginning and end of experiment and (2) between the red lines in (a).

The variation of the raw EE and IE and the calculated associated “apparent” Hct using the PV[O]H algorithm as the laser is scanned back and forth across the same trajectory on a healthy spinal cord are shown in Figs. 6(a) and 6(a). In Fig. 6(a), we display the raw IE and EE, i.e., contained in successive 20 ms frames, after having applied a 25-point adjacent average smoothing procedure.

We observe the average IE to decrease monotonically with some fluctuations either increasing or decreasing. In contrast, the EE is relatively constant on average but does fluctuate in a manner different from, but clearly correlated with, the IE fluctuations. This is analogous to observing the IE and EE at a single location but as a function of time, with fluctuations caused by, e.g., variations in perfusion. Here, we observe fluctuations due to variations in surface topography, and the composition of the cord is translated under the probe laser.

The bleaching can be inferred by the rise in “apparent Hct” at the hold locations.^10^ By the end of the experiment, the bleaching at position A, i.e., the amount of Hct, increases while holding at A is much less than at the beginning of the experiment also while holding at A for the same time interval. This behavior is exactly what we observe in skin with the bleaching being complete at a single location in about 5 to 10 min of continuous probing at these power levels.^10^ Once fully bleached, the tissue remains bleached for at least 5 h in actual live tissue, without additional exposure to the laser.^14^^,^^15^ This qualitative behavior of the probing/algorithm applied to spinal cord is essentially identical to that when applied to skin and despite the fact that we calibrated the algorithm in use against capillary blood Hct variation in skin, the same set of parameters a to f for Eq. (5) produces acceptable variation for image formation.^10^ That is, we do not expect the actual apparent Hct values to be meaningful, but the relative variation of “apparent” Hct as employed for imaging should be systematic, reproducible, and in fidelity with the actual physical appearance of the cord.

For comparing injured versus healthy spinal cords, we performed line scanning as described in Sec. 2 according to Fig. 3. These line scans of apparent Hct over time allow a methodical recording of fluorescence changes, increases in apparent turbidity, e.g., as might occur with bacterial infection of the CSF or the occurrence of excess protein in the CSF. The height of the surface of the animal changes less than the angle of incidence varies with the surface topography of the cord. Variation in distance between the laser aperture and the cord surface affects the spatial resolution of the image by changing the laser spot size. Visual observation suggests that in this study that variation is very small, probably less than 10%, i.e., the spot diameter might vary by between about 100 and 110  μm diameter. Inflammation, edema possibly, and other effects could induce similar changes over the course of time.

**Fig. 7:**
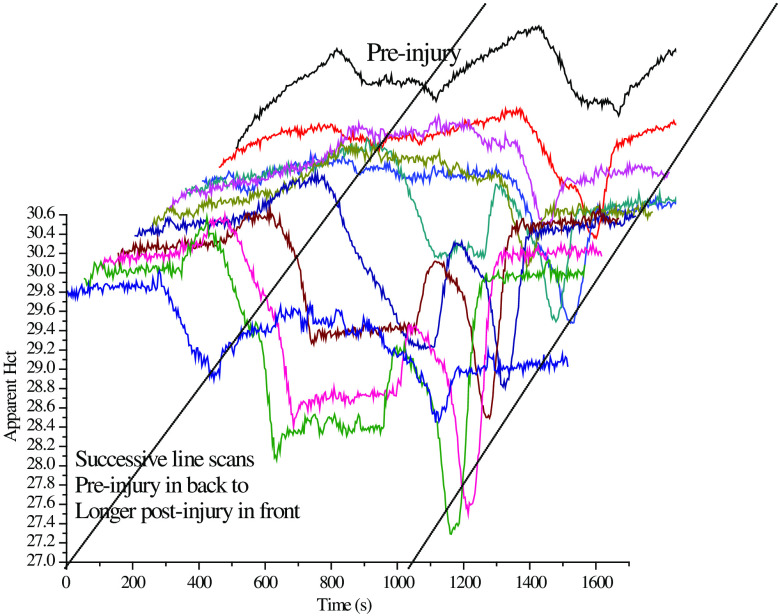
All 11 line scans from pre- to postinjury in a single SCI experiment. The time scale for scanning indicates the progression of the SCI, the bleaching process, and the position of the scan, because of the simultaneous bleaching that is occurring. Note that this is an “injury” experiment and “control” experiments were performed in exactly the same manner, including total time intervals, except no impaction/injury was performed. The preinjury scan is in the back with the successive scans in front. This is plotted so that the earliest scan is displayed translated slightly to the right and the later scans extend slightly to the left. Semi-log plots of IE for all scans at any particular Raman shift are essentially linear over the initial 5 h post-SCI including the preinjury scan.

At each hold location, we observe the Hct to increase, regardless of location, or whether the cord was injured or not, until the tissue is completely bleached. While in motion between locations, the Hct initially always decreases and then a variety of Hct responses are observed, including very sharp and deep dips such as near 1200 s in either the preinjured or first postinjury scans in Fig. 7. The Hct tends to decrease in between the reference locations because the tissue along the scan route bleaches more slowly than the tissue that receives continuous probing, i.e., the hold locations.

**Fig. 8:**
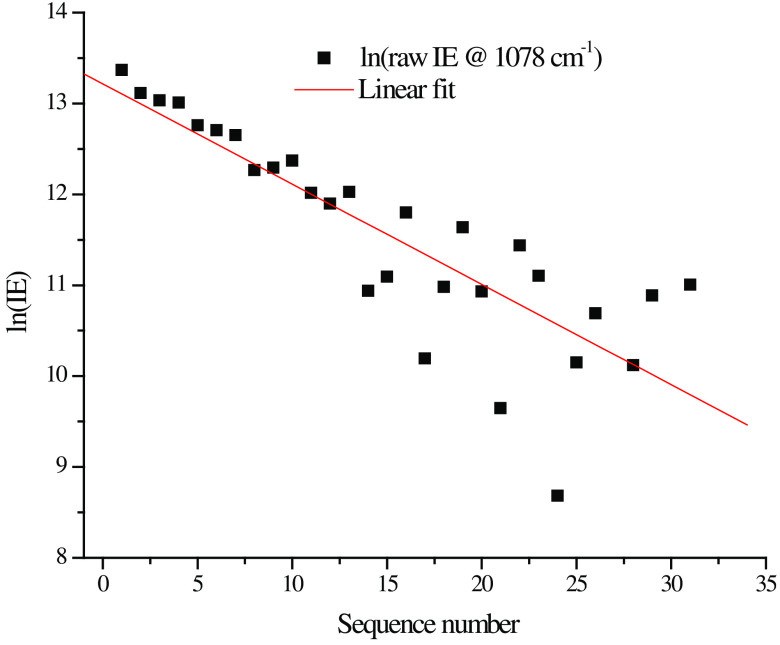
Semi-log plot of average IE for each of the 11 scans over the initial 5 h post-SCI including the preinjury scan.

The IE (total counts at Raman shift $1078\;{\mathrm{cm}}^{-1}$ including underlying fluorescence) collected for all 33 measurements were plotted semilogarithmically in Fig. 8. Despite the fact that the measurements combined results from three different hold locations, i.e., A, B, and C, the linearity of the result indicates that there is an exponential decay in IE during the data collection in the hold at each location, whereas the EE does not change appreciably. This reinforces our inference that autofluorescence photobleaching occurs and indicates that the spatial registration of the scanning system is reproducible and precise to within plus or minus the diameter of the laser spot ≈100  μm.

The same data were collected over the progression of the SCI initiated event; all 11 scans for this particular experiment are shown in Fig. 8. Note that as the number of successive scans increases the increase in Hct at each hold position diminishes indicating that whatever material is being bleached, it is not replaced in the time interval between successive scans. Thus, while there is almost certainly some emission from blood, i.e., from both RBCs and plasma, the bleached material is not blood since as long as the heart is beating, the blood is totally and continuously replaced in seconds from the probed volume.

The precipitous dip near 1200 s is unusual in that an equally precipitous rise quickly follows. The strong localization of the apparent Hct variation forces us to consider the possibility that we scanned over the blood vessel that can be seen in Fig. 4(a). We observed this type of structure in the PV[O]H line-scan image repeatedly, suggesting that PV[O]H images might have good fidelity with the actual appearance of the cord in each case. While we observe that the conditions for straightforward application of PV[O]H are usually met, as a localized event, scanning over a superficial vessel with relatively large vascular volume and Hct might not meet the requirements. Compared to other tissues, this probing would provide a unique mutual variation in EE and IE that is inconsistent with the PV[O]H assumptions for applicability, i.e., Eqs. (1) and (2) are obeyed and elastic scatterer density beyond the single scattering/linear propagation range.^9^ This could result in an uncharacteristic calculated value for turbidity/apparent Hct at those locations. Related to that consideration, we should also be cognizant that direct laser irradiation of blood in the intravascular space could result in excessive localized heating and ultimately, tissue damage.

**Fig. 9:**
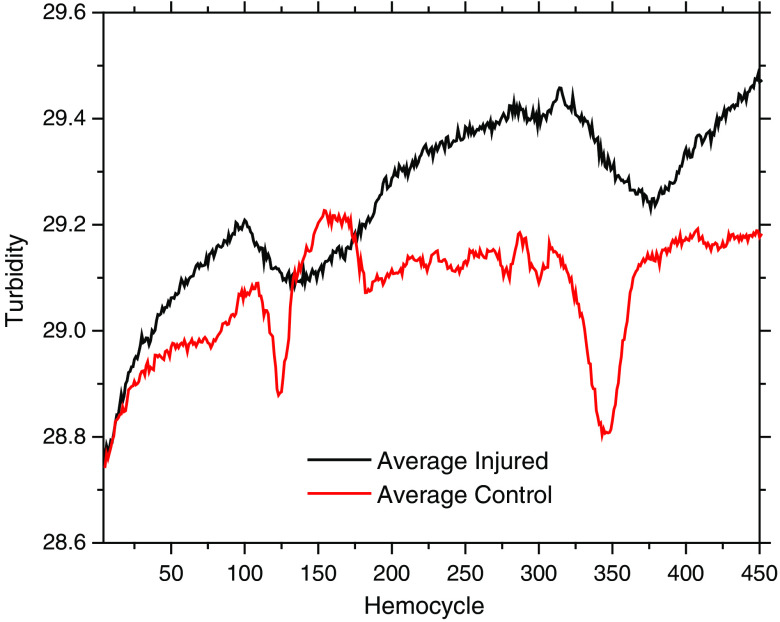
Line scans for averaged over three injured and three control animals. “Turbidity” is apparent Hct calculated from EE and IE using PV[O]H calibration. The injured animal scans display more bleaching effects while the control animals displayed more narrow and well-defined “dips.” Note: 1 hemocycle corresponds to 3 s.

Figure 9 shows the “average turbidity” line scans calculated by PV[O]H as a function of position(time) averaged over three separate injured rats and three separate control, i.e., uninjured rats and averaged over all scans in an experiment, i.e., sequence number. The sharper quality of the “dips” observed for the control animals as compared with the broader dips observed for the injured animals may result from registration errors in the scanning procedure. All animals were initially scanned before injury regardless of whether the animal was ultimately part of the injured or control cohort. The process of inducing injury did not involve additional movement to use the impactor specifically to avoid the opportunity for spatial registration error. So despite the small number of animals in each cohort, it is also possible that the difference in the average images results from cardiovascular changes induced by the injury itself.

### 3.2 Two-Dimensional Imaging

**Fig. 10:**
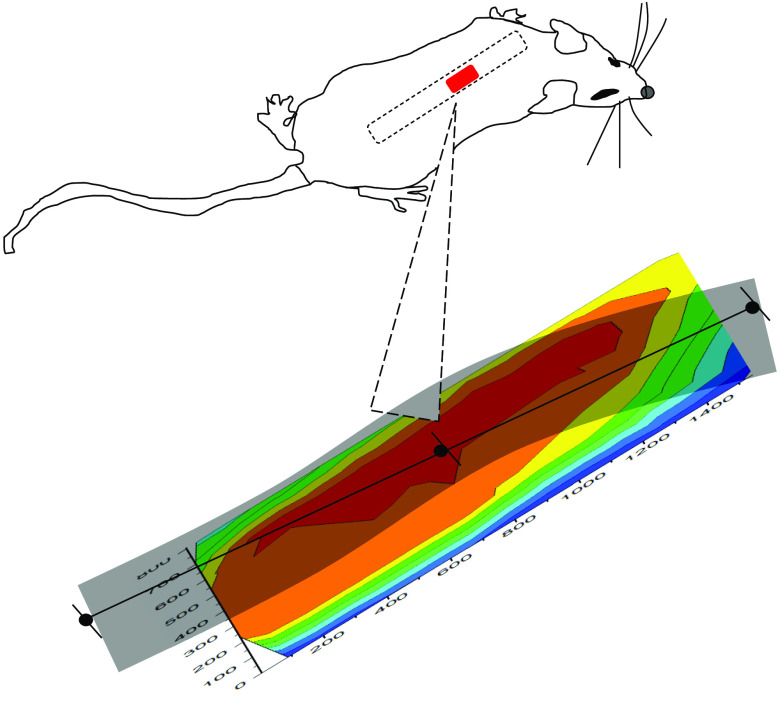
Image of surgical field including spinal cord based on Hct values obtained in a scan trajectory [Fig. 4(b)] designed so that no point is probed twice so as to avoid photobleaching effects from an early test scan causing systematic artifacts in later images. Note the systematic fall off of the apparent Hct on the edges of the image due at least in part because of topographic variation, i.e., curvature. The spatial scale is indicated on both axes using the time from the beginning of the first scan in seconds since the actual distances are as shown in Figs. 4(a) and 4(b), and the possibility of photobleaching artifacts is determined by the time duration of the laser exposure.

In order to explore the feasibility and possibly justify conducting a larger study, we performed a series of 2-D imaging experiments using one control and one injury model rat. The orientation and position of the surgical field and exposed spinal cord are shown in Fig. 10 along with an actual 2-D image. The orientation of all line scans, e.g., in Fig. 7 is shown in the black line and dots. The appearance in the images with rising and falling apparent Hct is independent of the position of scanning a specific location in the whole scan indicate that the choice of scan direction and total laser exposure per scan avoids photobleaching artifacts.

**Fig. 11:**
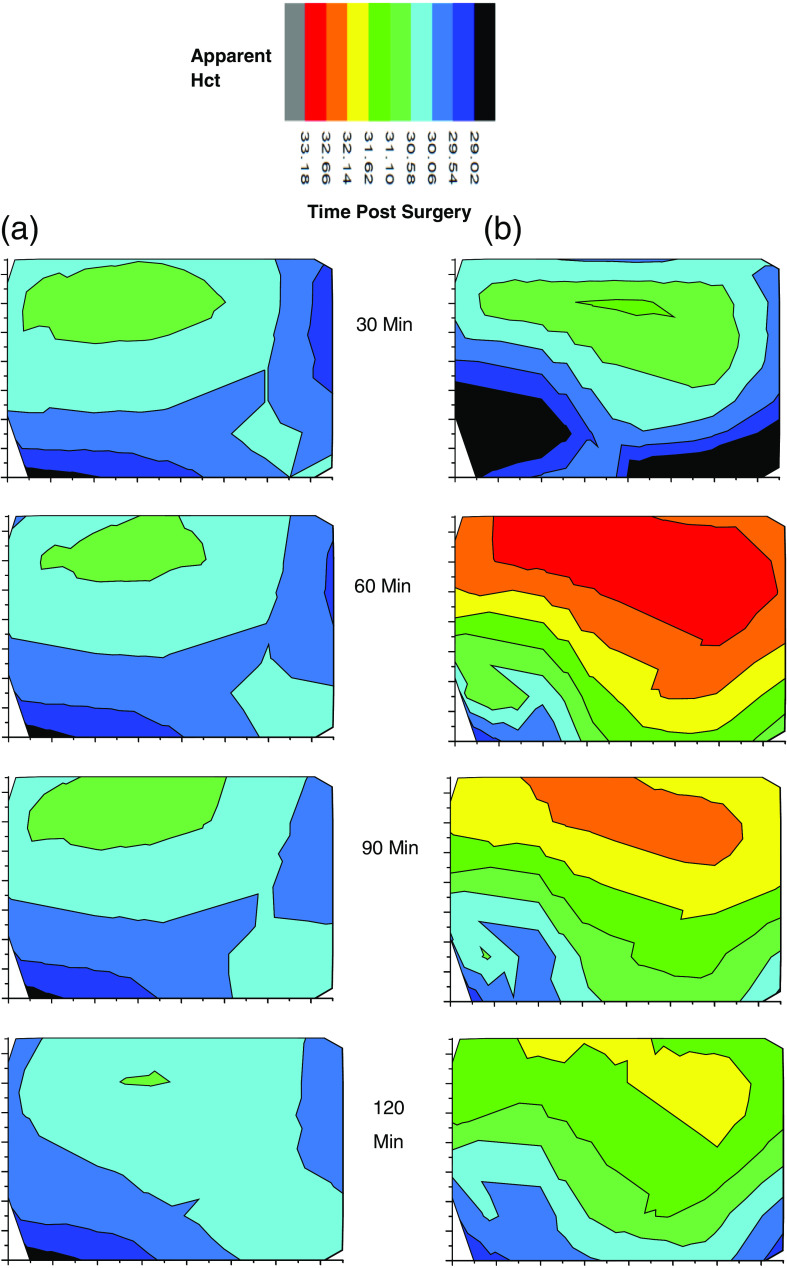
Successive images of (a) an uninjured cord and (b) an injured cord and at various times after surgery to exposed cord. The apparent Hct scale is the same for each image and the orientation and size of each image is as indicated in Fig. 10. Note that injury is not induced until after the first 30 min scan.

Successive images are shown in Fig. 11 for an injured and a control rat. There are notable differences but a definitive comparison must await additional data to possibly show statistical significance. Presently, we observe that the images of the probed regions of both animals may reflect the effect of injury. A larger apparent Hct usually involves increased IE and decreased EE. A topographical origin of such an observation could occur if there is greater curvature in the injured cord than in the control cord. Note that if incident probing, i.e., laser light, was maintained at a location of high apparent Hct for too long, bleaching would be observed if such was the case. As another possible cause, if there was swelling in the injured cord this would be observed.

Simulations based on the optical properties of cord tissue might reveal quantitatively how much change in apparent Hct could be associated with either effect. At this point, we cannot settle this question but we are encouraged that the images may in fact be classified based on the difference between internal physical and chemical status and topographical variation. It seems quite likely at this point that with more experience such difference could then be interpreted in terms of inflammation, edema, and/or swelling as one group of possibilities and bacterial infection or protein increase in the CSF as another. We note in passing that Raman spectra can be obtained at any location simultaneously with obtaining EE and IE and that would provide additional information to help classify and differentiate the possible causes and effects manifested in these images.

We note that the preinjury images of the two animals are essentially identical. And regardless of the above ambiguity, the successive images suggest that injury initiates processes that evolve over a period of >2  h from the time of injury. The images for the control animal are essentially constant over the entire experiment arguing that hydration is adequate and that the spatial scanning is also executed consistently.

**Fig. 12:**
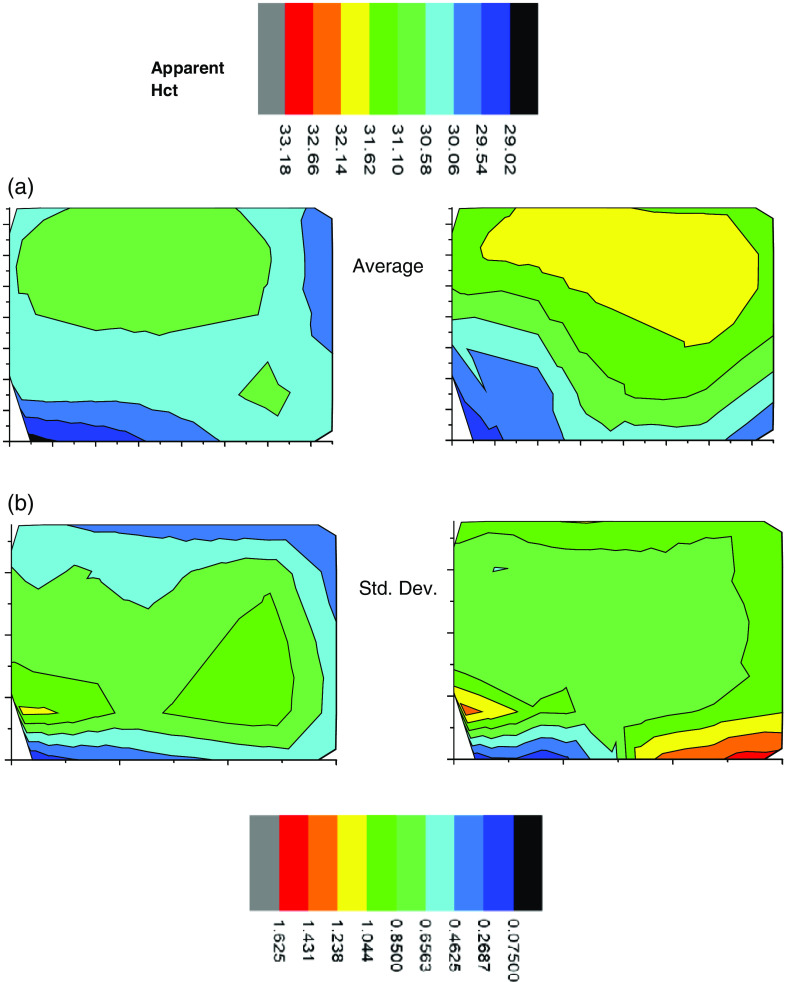
(a) Average of spinal cord images from Fig. 11 of both (left) uninjured and (right) uninjured cord over scanning time. (b) Standard deviation of spinal cord images of both (right) injured and (left) uninjured cord over scanning time.

Analogous to the line scans Fig. 12 shows the average 2-D image from the sequence shown in Fig. 11 as well as the standard deviation of each pixel. The standard deviation of a pixel in the center of the images is smaller by about a factor of 3 than the difference of the apparent Hct between control and injury in the same location.

We find the 2-D images to be remarkably consistent with the line scans. The average preinjury line scans start at a higher turbidity than the first 2-D image regardless of whether the rats are control or injured group. However, this is to be expected since care was taken throughout the 2-D scanning process to limit the exposure of the tissue to laser-induced bleaching. On the other hand, the line scans started at point A with a full 5 min exposure to laser probing, which caused photobleaching and the expected increase in measured turbidity. Furthermore, we note that the average 2-D images in Fig. 12 clearly show slightly greater apparent Hct, i.e., turbidity for the injured animal over the control animal averaged over all locations and timeframe. We also note that later 2-D images show turbidity increase near the edges at the later times, possibly because there was some drying out of the tissues. Uneven drying might also make the standard deviations of the calculated turbidity larger at longer times.

## 4 Discussion

Once accessed surgically, NIR probing of spinal cord *in vivo* is very much like probing other tissues. Given the profound insult required to surgically access the spinal cord, we were encouraged that surface blood and debris in the surgical field could be washed away significantly well to allow probing of the spinal cord itself. Experience shows that very little blood or any other absorbing chromophore is required to initiate burning, which was not observed in any of the experiments and results presented in this study.

As expected, the decay of the raw IE with increasing laser exposure to any one spot indicates that there is photobleaching of the cord. The behavior and timescale for the autofluorescence coming to equilibrium with the probing laser is similar to fingers, i.e., several minutes exposure at the power levels and spot size reported herein nearly completely bleaches a location. Also, pooled water or saline on the probed surface does not seem to alter the apparent Hct values. Since water does not fluoresce or have an appreciable Raman spectrum, this is not surprising. The internal consistency of multiple scanning experiments shows that it is easily possible to maintain spatial registration to at least ±100  μm during multihour experiments while maintaining hydration.

The line scans themselves suggest that such scanning produces images can be interpretable in terms of increasing/decreasing fluorescence, changing angle of incidence (surface topography), or combinations thereof. Assessing the behavior of autofluorescence or emission from exogenous fluorophores in the presence of turbidity, in a sensitive and reproducible manner, in response to procedures involving SCI or cords in general, is certainly a possibility using the PV[O]H algorithm. The actual capacity to assess such changes using PV[O]H images might be much better than we know from this study because in this study the calibration for the algorithm was based on capillaries in skin. Nevertheless, it produced variation in apparent Hct, i.e., turbidity that produced an image with good fidelity with the observed physical image. This suggesting that the basic spectroscopic and transport assumptions/requirements for the PV[O]H algorithm are met by spinal cord as well as by skin. We also wonder if a different calibration of PV[O]H specifically for SCI would produce even better performance.

Although much more data is needed, the actual results obtained in this study suggest that in the immediate aftermath of contusive injury, the most immediate consequence is an increase whatever causes increased apparent Hct. The first region, i.e., the subarachnoid space, encountered by probing photons after penetrating the dura contains fluid, i.e., CSF, and there are many possible interpretations of increased turbidity. Blood could actually leak into that space from damaged fine vessels or perhaps, some other fluorescent or more highly elastic scattering material leaks into the same space.

We will not settle this issue in this study, but it seems probable that we could make progress using PV[O]H in a larger study. In our one paired observation involving 2-D imaging, the increased apparent Hct dissipated over the succeeding 3 h after injury whereas the control animal was constant to ±1% over the same period. This corresponds to the movement of materials from the 100-μm diameter initial point of impact to about 1 mm in all directions over the succeeding 3 h. The observation demonstrates that it is possible to conduct the surgery to access the spinal cord in a manner that does not itself cause significant local physiological effect.

The issues of fluorescence and turbidity are particularly salient because as mentioned in Sec. 1, SCI and independent spinal cord infection could manifest by protein, viruses, or bacteria in the CSF. In this case, the turbidity and/or fluorescence yield of the CSF would change and this could be expected to affect the PV[O]H images. Given that CSF normally contains very little protein, PV[O]H might be ideal for differentiating bacterial infection from physical injury effects of spinal cords *in vivo*. To this point, future work might address the question of whether the spinal cord could be accessed for optical probing by placing a fiber optic inside a hypodermic needle that could be used to bring the probe next to but not touching the cord, as in an epidural anesthesia procedure. This seems like a viable approach at this time because the point data from line scans was in agreement with the 2-D images.

The abrupt dips observed in line scans were probably associated with near-surface blood vessels. This hypothesis is supported by the orientation of the dips relative to the scanning, their reproducibility, and comparison of the PV[O]H images with visual inspection. Since biomedical imaging can be very helpful if not essential to guide surgery or other interventions, being able to locate vessels and other points of reference is encouraging. Being able to locate such vessels also presents the possibility of testing the relative effect(s) of LLLT, or PBM involving direct probing of the vessels or the surrounding tissues.^6^

Finally, there are variations on the PV[O]H algorithm, e.g., involving simultaneous Raman spectra as the IE, to produce images that could relate blood presence, fluid presence, localized inflammation, or hypoxic conditions.^4^ We will present Raman spectroscopic results from this study separately but presently we believe that unique internal chemical/physical analysis from spectroscopic/physical probing can be obtained without contact. We suggest that such imaging can be integrated with information or images from other devices, e.g., robotic surgical suites to guide treatment.

## 5 Conclusions

PV[O]H images of exposed spinal cord *in vivo* obtained during the immediate locale/aftermath of moderate contusive injury reveal apparent changes in turbidity and/or fluorescence in the CSF that are different from control. These differences dissipate in the succeeding 3 h. We have employed the PV[O]H algorithm to (1) create 1- and 2-D images, (2) locate near surface blood vessels, and (3) show that spinal cord tissue photobleaches in a manner similar to skin.
